# Supplementary material for: Adverse outcomes after partner bereavement in people with reduced kidney function: Parallel cohort studies in England and Denmark
Source: PLoS One. 2021 Sep 23;16(9):e0257255. doi: 10.1371/journal.pone.0257255 (PMC8460004; doi:10.1371/journal.pone.0257255)
Supplement: S1 Table — (DOCX) [file pone.0257255.s001.docx]

### **S1 Table.** Association between partner bereavement and study outcomes stratified by age group, sex, and CKD stage.

|  |  |  | **UK** | | | **DK** | | |
| --- | --- | --- | --- | --- | --- | --- | --- | --- |
| **Outcome** | **Stratifier** | **Stratified group** | **Events in bereaved** | **Unadjusted HR** | **Adjusted HR*** | **Events in bereaved** | **Unadjusted HR** | **Adjusted HR*** |
| Composite CVD | Overall | - | 2621 | 1.06 (1.01-1.11) | 1.06 (1.01-1.12) | 1494 | 1.12 (1.06-1.18) | 1.10 (1.04-1.17) |
|  | Age group | <65 | 45 | 1.55 (1.07-2.26) | 1.38 (0.93-2.05) | 203 | 1.31 (1.12-1.52) | 1.26 (1.06-1.49) |
|  |  | 65-74 | 441 | 1.20 (1.08-1.34) | 1.18 (1.05-1.32) | 505 | 1.19 (1.07-1.31) | 1.09 (0.98-1.22) |
|  |  | 75+ | 2135 | 1.03 (0.97-1.08) | 1.03 (0.98-1.09) | 786 | 1.04 (0.96-1.13) | 1.07 (0.98-1.16) |
|  | Sex | Male | 1093 | 1.11 (1.03-1.19) | 1.12 (1.04-1.21) | 780 | 1.10 (1.02-1.18) | 1.10 (1.02-1.19) |
|  |  | Female | 1528 | 1.03 (0.97-1.09) | 1.02 (0.96-1.09) | 714 | 1.14 (1.05-1.24) | 1.09 (1.00-1.19) |
|  | CKD stage | 3a | 1617 | 1.10 (1.04-1.68) | 1.10 (1.03-1.17) | - | - | - |
|  |  | 3b | 779 | 1.00 (0.91-1.10) | 1.02 (0.93-1.13) | - | - | - |
|  |  | 4-5 | 225 | 0.91 (0.76-1.09) | 0.92 (0.76-1.11) | - | - | - |
| AKI | Overall | - | 1136 | 1.18 (1.10-1.27) | 1.20 (1.10-1.31) | 246 | 1.40 (1.21-1.62) | 1.36 (1.17-1.58) |
|  | Age group | <65 | 24 | 1.75 (1.03-2.98) | 1.72 (0.98-3.04) | 54 | 1.65 (1.20-2.27) | 1.35 (0.95-1.93) |
|  |  | 65-74 | 192 | 1.18 (1.00-1.40) | 1.12 (0.94-1.34) | 94 | 1.66 (1.32-2.09) | 1.55 (1.22-1.97) |
|  |  | 75+ | 920 | 1.17 (1.08-1.27) | 1.21 (1.11-1.32) | 98 | 1.18 (0.93-1.48) | 1.22 (0.96-1.55) |
|  | Sex | Male | 506 | 1.24 (1.11-1.38) | 1.25 (1.11-1.40) | 125 | 1.50 (1.24-1.82) | 1.48 (1.21-1.81) |
|  |  | Female | 630 | 1.14 (1.03-1.26) | 1.16 (1.04-1.29) | 121 | 1.30 (1.05-1.61) | 1.23 (0.99-1.53) |
|  | CKD stage | 3a | 597 | 1.24 (1.12-1.37) | 1.22 (1.10-1.36) | - | - | - |
|  |  | 3b | 412 | 1.20 (1.04-1.37) | 1.20 (1.04-1.38) | - | - | - |
|  |  | 4-5 | 127 | 1.08 (0.82-1.41) | 1.09 (0.82-1.44) | - | - | - |
| Death | Overall | - | 6135 | 1.12 (1.08-1.15) | 1.10 (1.05-1.14) | 2809 | 1.20 (1.15-1.25) | 1.20 (1.15-1.25) |
|  | Age group | <65 | 81 | 1.50 (1.14-1.97) | 1.43 (1.07-1.91) | 271 | 1.39 (1.21-1.60) | 1.25 (1.07-1.45) |
|  |  | 65-74 | 863 | 1.25 (1.15-1.35) | 1.18 (1.09-1.28) | 886 | 1.28 (1.18-1.38) | 1.26 (1.16-1.38) |
|  |  | 75+ | 5191 | 1.09 (1.05-1.13) | 1.08 (1.04-1.12) | 1652 | 1.13 (1.07-1.20) | 1.16 (1.09-1.23) |
|  | Sex | Male | 2669 | 1.14 (1.09-1.20) | 1.13 (1.08-1.19) | 1453 | 1.19 (1.12-1.26) | 1.20 (1.13-1.27) |
|  |  | Female | 3466 | 1.10 (1.05-1.15) | 1.07 (1.03-1.12) | 1356 | 1.21 (1.13-1.29) | 1.20 (1.13-1.29) |
|  | CKD stage | 3a | 3690 | 1.14 (1.09-1.19) | 1.12 (1.07-1.16) | - | - | - |
|  |  | 3b | 1847 | 1.08 (1.02-1.15) | 1.09 (1.02-1.16) | - | - | - |
|  |  | 4-5 | 598 | 1.04 (0.92-1.17) | 1.00 (0.88-1.13) | - | - | - |
|  |  |  |  |  |  |  |  |  |
| *England: adjusted for comorbidities (CKD stage, cerebrovascular disease, heart failure, chronic obstructive pulmonary disease, diabetes, hypertension, ischaemic heart disease, myocardial infarction, peripheral artery disease, connective tissue disease, dementia, peptic ulcers, non-haematological cancer, haematological cancer, liver disease), history of AKI, smoking status, alcohol consumption, BMI category, IMD category  *Denmark: adjusted for comorbidities (cerebrovascular disease, heart failure, chronic obstructive pulmonary disease, diabetes, hypertension, ischaemic heart disease, myocardial infarction, peripheral artery disease, connective tissue disease, dementia, peptic ulcers, non-haematological cancer, haematological cancer, liver disease), history of AKI, and educational attainment. | | | | | | | | |
