# Supplementary material for: Adverse outcomes after partner bereavement in people with reduced kidney function: Parallel cohort studies in England and Denmark
Source: PLoS One. 2021 Sep 23;16(9):e0257255. doi: 10.1371/journal.pone.0257255 (PMC8460004; doi:10.1371/journal.pone.0257255)
Supplement: S2 Table — (DOCX) [file pone.0257255.s002.docx]

### **S2 Table.** Risk of CVD, AKI, and death in persons with CKD with or without bereavement restricting study period to 2010-2018 (England only)

| Population | Outcome | Bereaved cohort | | | Comparison cohort | | | Unadjusted HR (95% CI) | Adjusted HR* (95% CI) |
| --- | --- | --- | --- | --- | --- | --- | --- | --- | --- |
|  |  | Number of events | Person years at-risk | Rate per 1,000 | Number of events | Person years at-risk | Rate per 1,000 |  |  |
|  |  |  |  |  |  |  |  |  |  |
| UK | CVD | 610 | 17133 | 35.6 (32.9-38.5) | 3376 | 109598 | 30.8 (29.8-31.9) | 1.06 (1.01-1.11) | 1.06 (1.01-1.11) |
|  | AKI | 408 | 17433 | 23.4 (21.2-25.8) | 1952 | 111633 | 17.5 (16.7-18.3) | 1.18 (1.10-1.27) | 1.20 (1.11-1.30) |
|  | Death | 1351 | 17884 | 75.5 (71.6-79.7) | 6948 | 113488 | 61.2 (59.8-62.7) | 1.12 (1.08-1.15) | 1.10 (1.06-1.13) |
| *Adjusted for comorbidities (CKD stage, cerebrovascular disease, heart failure, chronic obstructive pulmonary disease, diabetes, hypertension, ischaemic heart disease, myocardial infarction, peripheral artery disease, connective tissue disease, dementia, peptic ulcers, non-haematological cancer, haematological cancer, liver disease), history of AKI, smoking status, alcohol consumption, BMI category, IMD category | | | | | | | | | |
