# Supplementary material for: Adverse outcomes after partner bereavement in people with reduced kidney function: Parallel cohort studies in England and Denmark
Source: PLoS One. 2021 Sep 23;16(9):e0257255. doi: 10.1371/journal.pone.0257255 (PMC8460004; doi:10.1371/journal.pone.0257255)
Supplement: S3 Table — (DOCX) [file pone.0257255.s003.docx]

### **S3 Table.** Sensitivity analysis – repeat main analysis in English cohort using matching without replacement

| Population | Outcome | Bereaved cohort | | | Comparison cohort | | | Unadjusted HR  (95% CI) | Adjusted HR* (95% CI) |
| --- | --- | --- | --- | --- | --- | --- | --- | --- | --- |
|  |  | Number of events | Person years at-risk | Rate per 1,000 | Number of events | Person years at-risk | Rate per 1,000 |  |  |
|  |  |  |  |  |  |  |  |  |  |
| UK | **Composite CVD** | 1730 | 56086 | 30.8 (29.4-32.3) | 3947 | 137909 | 28.6 (27.7-29.5) | 1.09 (1.02-1.17) | 1.10 (1.02-1.18) |
|  | Heart failure | 931 | 57748 | 16.1 (15.1-17.2) | 2086 | 141562 | 14.7 (14.1-15.4) | 1.14 (1.04-1.25) | 1.16 (1.04-1.30) |
|  | Myocardial infarction | 462 | 58350 | 7.92 (7.23-8.67) | 1122 | 142677 | 7.83 (7.42-8.34) | 1.05 (0.92-1.19) | 1.02 (0.89-1.17) |
|  | Stroke | 562 | 58352 | 9.63 (8.87-10.5) | 1272 | 142966 | 8.90 (8.42-9.40) | 1.06 (0.94-1.19) | 1.08 (0.95-1.23) |
|  | **AKI** | 720 | 58391 | 12.3 (11.5-13.3) | 1414 | 143331 | 9.87 (9.36-10.4) | 1.18 (1.05-1.31) | 1.17 (1.04-1.32) |
|  | **Death** | 3940 | 59351 | 66.4 (64.3-68.5) | 8407 | 145001 | 58.0 (56.8-59.2) | 1.14 (1.09-1.19) | 1.12 (1.07-1.18) |
| *Adjusted for comorbidities (CKD stage, cerebrovascular disease, heart failure, chronic obstructive pulmonary disease, diabetes, hypertension, ischaemic heart disease, myocardial infarction, peripheral artery disease, connective tissue disease, dementia, peptic ulcers, non-haematological cancer, haematological cancer, liver disease), history of AKI, smoking status, alcohol consumption, BMI category, IMD category | | | | | | | | | |
