# Supplementary material for: Adverse outcomes after partner bereavement in people with reduced kidney function: Parallel cohort studies in England and Denmark
Source: PLoS One. 2021 Sep 23;16(9):e0257255. doi: 10.1371/journal.pone.0257255 (PMC8460004; doi:10.1371/journal.pone.0257255)
Supplement: S4 Table — (DOCX) [file pone.0257255.s004.docx]

### **S4 Table.** Risk of CVD in person with CKD with or without bereavement in England and Denmark stratified by follow-up periods

| Population | Follow-up period | Bereaved cohort | | | Comparison cohort | | | Unadjusted HR (95% CI) | Adjusted HR* (95% CI) |
| --- | --- | --- | --- | --- | --- | --- | --- | --- | --- |
|  |  | Number of events | Person years at-risk | Rate per 1,000 | Number of events | Person years at-risk | Rate per 1,000 |  |  |
|  |  |  |  |  |  |  |  |  |  |
| UK | 0-1 years | 661 | 17576 | 37.6 (34.8-40.6) | 3568 | 121847 | 29.3 (28.3-30.3) | 1.17 (1.07-1.28) | 1.20 (1.09-1.32) |
|  | 0-2 years | 1101 | 31816 | 34.6 (32.6-36.7) | 6261 | 220467 | 28.4 (27.7-29.1) | 1.11 (1.04-1.19) | 1.12 (1.05-1.21) |
|  | 0-3 years | 1460 | 43513 | 33.6 (31.9-35.3) | 8370 | 299836 | 27.9 (27.3-28.5) | 1.10 (1.04-1.17) | 1.11 (1.04-1.18) |
|  | 0-4 years | 1725 | 53092 | 32.5 (31.0-34.1) | 10070 | 36293 | 27.7 (27.2-28.3) | 1.08 (1.02-1.14) | 1.08 (1.02-1.15) |
|  | 0-5 years | 1952 | 60841 | 32.1 (30.7-33.5) | 11361 | 412253 | 27.6 (27.1-28.1) | 1.08 (1.03-1.14) | 1.08 (1.03-1.14) |
|  | Complete follow-up | 2621 | 82747 | 31.7 (30.5-32.9) | 14942 | 538165 | 27.8 (27.3-28.2) | 1.06 (1.01-1.11) | 1.06 (1.01-1.12) |
|  |  |  |  |  |  |  |  |  |  |
| DK | 0-1 years | 562 | 4,535 | 123.9 (114.0-134.5) | 3,350 | 30,053 | 111.5 (107.7-115.3) | 1.14 (1.05-1.24) | 1.15 (1.05-1.26) |
|  | 0-2 years | 831 | 7,916 | 105.0 (98.0-112.3) | 5,061 | 51,904 | 97.5 (94.8-100.2) | 1.10 (1.03-1.18) | 1.10 (1.02-1.19) |
|  | 0-3 years | 1,010 | 10,536 | 95.9 (90.1-101.9) | 6,123 | 67,855 | 90.2 (88.0-92.5) | 1.09 (1.02-1.17) | 1.08 (1.01-1.16) |
|  | 0-4 years | 1,139 | 12,557 | 90.7 (85.6-96.1) | 6,831 | 79,540 | 85.9 (83.9-87.9) | 1.09 (1.02-1.16) | 1.08 (1.01-1.15) |
|  | 0-5 years | 1,227 | 14,111 | 87.0 (82.2-91.9) | 7,291 | 87,953 | 82.9 (81.0-84.8) | 1.09 (1.03-1.15) | 1.08 (1.01-1.15) |
|  | Complete follow-up | 1,494 | 18,962 | 78.8 (74.9-82.9) | 8,265 | 110,315 | 74.9 (73.3-76.6) | 1.12 (1.06-1.18) | 1.10 (1.04-1.17) |
| *England: adjusted for comorbidities (CKD stage, cerebrovascular disease, heart failure, chronic obstructive pulmonary disease, diabetes, hypertension, ischaemic heart disease, myocardial infarction, peripheral artery disease, connective tissue disease, dementia, peptic ulcers, non-haematological cancer, haematological cancer, liver disease), history of AKI, smoking status, alcohol consumption, BMI category, IMD category  *Denmark: adjusted for comorbidities (cerebrovascular disease, heart failure, chronic obstructive pulmonary disease, diabetes, hypertension, ischaemic heart disease, myocardial infarction, peripheral artery disease, connective tissue disease, dementia, peptic ulcers, non-haematological cancer, haematological cancer, liver disease), history of AKI, and educational attainment. | | | | | | | | | |
