# Supplementary material for: Adverse outcomes after partner bereavement in people with reduced kidney function: Parallel cohort studies in England and Denmark
Source: PLoS One. 2021 Sep 23;16(9):e0257255. doi: 10.1371/journal.pone.0257255 (PMC8460004; doi:10.1371/journal.pone.0257255)
Supplement: S5 Table — (DOCX) [file pone.0257255.s005.docx]

### **S5 Table.** Risk of AKI in person with CKD with or without bereavement in England and Denmark stratified by follow-up periods

| Population | Follow-up period | Bereaved cohort | | | Comparison cohort | | | Unadjusted HR  (95% CI) | Adjusted HR*  (95% CI) |
| --- | --- | --- | --- | --- | --- | --- | --- | --- | --- |
|  |  | Number of events | Person years at-risk | Rate per 1,000 | Number of events | Person years at-risk | Rate per 1,000 |  |  |
|  |  |  |  |  |  |  |  |  |  |
| UK | 0-1 years | 208 | 17739 | 11.7 (10.2-13.4) | 974 | 122846 | 7.93 (7.45-8.44) | 1.32 (1.13-1.54) | 1.37 (1.15-1.63) |
|  | 0-2 years | 362 | 32295 | 11.2 (10.1-12.4) | 1842 | 223551 | 8.24 (7.87-8.62) | 1.21 (1.08-1.36) | 1.27 (1.11-1.44) |
|  | 0-3 years | 498 | 44373 | 11.2 (10.3-12.3) | 2578 | 305450 | 8.44 (8.12-8.77) | 1.21 (1.09-1.34) | 1.24 (1.11-1.39) |
|  | 0-4 years | 639 | 54343 | 11.8 (10.9-12.7) | 3205 | 371215 | 8.63 (8.34-8.94) | 1.22 (1.11-1.34) | 1.25 (1.13-1.38) |
|  | 0-5 years | 736 | 62475 | 11.8 (11.0-12.7) | 3723 | 423007 | 8.80 (8.52-9.09) | 1.19 (1.09-1.29) | 1.22 (1.11-1.34) |
|  | Complete follow-up | 1136 | 85950 | 13.2 (12.5-14.0) | 5560 | 557977 | 9.96 (9.71-10.2) | 1.18 (1.10-1.27) | 1.20 (1.10-1.31) |
|  |  |  |  |  |  |  |  |  |  |
| DK | 0-1 years | 76 | 4,732 | 16.1 (12.7-20.0) | 334 | 31,321 | 10.7 (9.6-11.9) | 1.71 (1.34-2.18) | 1.73 (1.34-2.22) |
|  | 0-2 years | 120 | 8,469 | 14.2 (11.8-16.9) | 585 | 55,301 | 10.6 (9.7-11.5) | 1.53 (1.27-1.85) | 1.52 (1.25-1.85) |
|  | 0-3 years | 147 | 11,462 | 12.8 (10.9-15.0) | 738 | 73,504 | 10.0 (9.3-10.8) | 1.47 (1.23-1.74) | 1.47 (1.23-1.75) |
|  | 0-4 years | 168 | 13,849 | 12.1 (10.4-14.1) | 844 | 87,203 | 9.7 (9.0-10.3) | 1.46 (1.24-1.72) | 1.44 (1.22-1.71) |
|  | 0-5 years | 186 | 15,726 | 11.8 (10.2-13.6) | 927 | 97,310 | 9.5 (8.9-10.2) | 1.42 (1.21-1.66) | 1.38 (1.18-1.62) |
|  | Complete follow-up | 246 | 21,925 | 11.2 (9.9-12.7) | 1,157 | 125,022 | 9.3 (8.7-9.8) | 1.40 (1.21-1.62) | 1.36 (1.17-1.58) |
| *England: adjusted for comorbidities (CKD stage, cerebrovascular disease, heart failure, chronic obstructive pulmonary disease, diabetes, hypertension, ischaemic heart disease, myocardial infarction, peripheral artery disease, connective tissue disease, dementia, peptic ulcers, non-haematological cancer, haematological cancer, liver disease), history of AKI, smoking status, alcohol consumption, BMI category, IMD category  *Denmark: adjusted for comorbidities (cerebrovascular disease, heart failure, chronic obstructive pulmonary disease, diabetes, hypertension, ischaemic heart disease, myocardial infarction, peripheral artery disease, connective tissue disease, dementia, peptic ulcers, non-haematological cancer, haematological cancer, liver disease), history of AKI, and educational attainment. | | | | | | | | | |
