# Supplementary material for: Adverse outcomes after partner bereavement in people with reduced kidney function: Parallel cohort studies in England and Denmark
Source: PLoS One. 2021 Sep 23;16(9):e0257255. doi: 10.1371/journal.pone.0257255 (PMC8460004; doi:10.1371/journal.pone.0257255)
Supplement: S6 Table — (DOCX) [file pone.0257255.s006.docx]

### **S6 Table.** Risk of death in person with CKD with or without bereavement in England and Denmark stratified by follow-up periods

| Population | Follow-up period | Bereaved cohort | | | Comparison cohort | | | Unadjusted HR  (95% CI) | Adjusted HR*  (95% CI) |
| --- | --- | --- | --- | --- | --- | --- | --- | --- | --- |
|  |  | Number of events | Person years at-risk | Rate per 1,000 | Number of events | Person years at-risk | Rate per 1,000 |  |  |
|  |  |  |  |  |  |  |  |  |  |
| UK | 0-1 years | 1274 | 17811 | 71.5 (67.7-75.6) | 6103 | 123160 | 49.6 (48.3-50.8) | 1.28 (1.20-1.36) | 1.27 (1.19-1.37) |
|  | 0-2 years | 2186 | 32510 | 67.2 (64.5-70.1) | 11287 | 224521 | 50.3 (49.4-51.2) | 1.19 (1.13-1.24) | 1.17 (1.11-1.23) |
|  | 0-3 years | 2994 | 44758 | 66.9 (64.5-69.3) | 15740 | 307250 | 51.2 (50.4-52.0) | 1.16 (1.11-1.21) | 1.15 (1.10-1.20) |
|  | 0-4 years | 3649 | 54912 | 66.5 (64.3-68.6) | 19410 | 373904 | 51.9 (51.2-52.6) | 1.14 (1.10-1.18) | 1.13 (1.09-1.18) |
|  | 0-5 years | 4179 | 63218 | 66.1 (64.1-68.1) | 22399 | 426508 | 52.5 (51.8-53.2) | 1.12 (1.08-1.17) | 1.11 (1.07-1.15) |
|  | Complete follow-up | 6135 | 87389 | 70.2 (68.5-72.0) | 31194 | 564437 | 55.3 (54.7-55.9) | 1.12 (1.08-1.15) | 1.10 (1.05-1.14) |
|  |  |  |  |  |  |  |  |  |  |
| DK | 0-1 years | 756 | 4,758 | 158.9 (147.9-170.5) | 3,755 | 31,429 | 119.5 (115.7-123.3) | 1.38 (1.28-1.48) | 1.39 (1.29-1.51) |
|  | 0-2 years | 1,230 | 8,538 | 144.1 (136.2-152.3) | 6,499 | 55,612 | 116.9 (114.0-119.7) | 1.28 (1.20-1.35) | 1.29 (1.21-1.37) |
|  | 0-3 years | 1,558 | 11,577 | 134.6 (128.0-141.4) | 8,510 | 74,025 | 115.0 (112.5-117.4) | 1.21 (1.14-1.27) | 1.21 (1.15-1.28) |
|  | 0-4 years | 1,845 | 14,004 | 131.7 (125.8-137.9) | 10,035 | 87,914 | 114.1 (111.9-116.4) | 1.21 (1.15-1.27) | 1.21 (1.15-1.28) |
|  | 0-5 years | 2,075 | 15,911 | 130.4 (124.9-136.1) | 11,124 | 98,191 | 113.3 (111.2-115.4) | 1.20 (1.14-1.25) | 1.20 (1.14-1.26) |
|  | Complete follow-up | 2,809 | 22,229 | 126.4 (121.8-131.1) | 13,956 | 126,523 | 110.3 (108.5-112.1) | 1.20 (1.15-1.25) | 1.20 (1.15-1.25) |
| *England: adjusted for comorbidities (CKD stage, cerebrovascular disease, heart failure, chronic obstructive pulmonary disease, diabetes, hypertension, ischaemic heart disease, myocardial infarction, peripheral artery disease, connective tissue disease, dementia, peptic ulcers, non-haematological cancer, haematological cancer, liver disease), history of AKI, smoking status, alcohol consumption, BMI category, IMD category  *Denmark: adjusted for comorbidities (cerebrovascular disease, heart failure, chronic obstructive pulmonary disease, diabetes, hypertension, ischaemic heart disease, myocardial infarction, peripheral artery disease, connective tissue disease, dementia, peptic ulcers, non-haematological cancer, haematological cancer, liver disease), history of AKI, and educational attainment. | | | | | | | | | |
