# Supplementary material for: Adverse outcomes after partner bereavement in people with reduced kidney function: Parallel cohort studies in England and Denmark
Source: PLoS One. 2021 Sep 23;16(9):e0257255. doi: 10.1371/journal.pone.0257255 (PMC8460004; doi:10.1371/journal.pone.0257255)
Supplement: S1 Methods — (DOCX) [file pone.0257255.s007.docx]

### **S1 Methods. Data sources – England**

The Clinical Practice Research Datalink (CPRD) Gold version^28^ is a primary care dataset which covers approximately 7% of the UK population. It contains primary care diagnoses (coded using Read codes), prescriptions, and laboratory test results for patients registered at contributing General Practices (GPs). We obtained bereavement (exposure) status, communal living status, serum creatinine measures (used to derive estimated glomerular filtration (eGFR) and CKD catgory), age, sex, comorbidity status, history of AKI or renal replacement therapy, smoking stutus, alcohol consumption, and body-mass index (BMI) from CPRD data.

Hospital Episode Statistics (HES) secondary care data^42^ contain information on admission and discharge dates, and diagnoses recorded using International Classification of Diseases 10^th^ edition (ICD-10) codes for all National Health Service (NHS)-funded patients in England. We used these data to identify outcomes, as well as to identify comorbidities (in addition to primary care data).

Office of National Statistics (ONS) data provides dates and causes of death for all deaths recorded in England. We used these data to define bereavement (exposure) status, as well as to define the mortality outcome.

The Index of Multiple Deprivation (IMD) is a deprivation index used as a proxy for socioeconomic status (SES) in England which groups people into deprivation quintiles based on the person’s postcode, or the general practice (GP) postcode when the person’s postcode is missing. We used this index as a proxy for socioeconomic status.
