# Supplementary material for: Adverse outcomes after partner bereavement in people with reduced kidney function: Parallel cohort studies in England and Denmark
Source: PLoS One. 2021 Sep 23;16(9):e0257255. doi: 10.1371/journal.pone.0257255 (PMC8460004; doi:10.1371/journal.pone.0257255)
Supplement: S2 Methods — (DOCX) [file pone.0257255.s008.docx]

### **S2 Methods. Data sources – Denmark**

*The Danish Civil Registration System*,^29^ holding personal identification numbers, *i.e.*, CPR numbers, a unique 10-digit number for every Danish resident given at birth or immigration. CPR numbers enables linking individuals across the nationwide registries. The registry also holds information on date of birth, sex, vital status, CPR numbers of spouse, parents and children, and place of living, of every person in Denmark.

*The Danish National Patient Registry*,^30^ including information on all in- and outpatient hospital contacts since 1995, with details about date of admission and discharge, procedures and operations, and one or more discharge diagnoses. The discharge diagnoses are recorded using the International Classification of Diseases, Eighth Revision (ICD-8) until 1994, and the International Classification of Diseases, Tenth Revision (ICD-10) thereafter.

*The Danish Education Registers*,^32^ including information on the highest completed level of education for each individual. In persons born from 1945 through 1990, information on educational attainment is available for 97% of ethnic Danes, and 85–90% of immigrants. In persons born before 1945, are larger proportion has missing information on educational attainment.
