# Supplementary material for: Adverse outcomes after partner bereavement in people with reduced kidney function: Parallel cohort studies in England and Denmark
Source: PLoS One. 2021 Sep 23;16(9):e0257255. doi: 10.1371/journal.pone.0257255 (PMC8460004; doi:10.1371/journal.pone.0257255)
Supplement: S3 Methods — (DOCX) [file pone.0257255.s009.docx]

### **S3 Methods. Partner identification – England**

The partner algorithm is based on the CPRD family number, which identifies people living in the same household on the study initiation date. Among people with the same family number, we identified couples as two individuals of the opposite sex, with an age gap of ≤10 years and with no younger adult in the household within 15 years of either of the couple (to avoid misclassifying co-habiting friends or flatmates as romantic partners). In addition, we excluded cohabitees where a person in the couple had any code indicating residence in a communal establishment before the latest of practice registration date or study initiation date, both individuals in the couple were <40 years or ≥95 years old, or the family number was used for >10 people.
