# Supplementary material for: Adverse outcomes after partner bereavement in people with reduced kidney function: Parallel cohort studies in England and Denmark
Source: PLoS One. 2021 Sep 23;16(9):e0257255. doi: 10.1371/journal.pone.0257255 (PMC8460004; doi:10.1371/journal.pone.0257255)
Supplement: S4 Methods — (DOCX) [file pone.0257255.s010.docx]

### **S4 Methods. Partner identification – Denmark**

This partner algorithm combines data on civil status, demographics, address, and close kinship to identify a deceased person’s partner. The algorithm allows for up to a 15-year gap between partners because more detailed data relevant to partner status are available.
