# Supplementary material for: Adverse outcomes after partner bereavement in people with reduced kidney function: Parallel cohort studies in England and Denmark
Source: PLoS One. 2021 Sep 23;16(9):e0257255. doi: 10.1371/journal.pone.0257255 (PMC8460004; doi:10.1371/journal.pone.0257255)
Supplement: S5 Methods — (DOCX) [file pone.0257255.s011.docx]

### **S5 Methods. Lifestyle risk factor algorithms – England**

| Smoking algorithm | We searched each person’s CPRD clinical and additional files for Read codes indicating smoking status, CPRD therapy files for nicotine replacement therapy prescriptions, and HES secondary care data for ICD-10 codes indicating smoking status. We combined these data and created three levels of smoking status: (1) non-smoker, (2) ex-smoker, (3) current smoker. We recoded people whose most recent smoking status indicated non-smoker, but had a history of smoking as ex-smokers.  We prioritised data indicating smoking status in the following order:  1. Smoking record within -365 to + 30 days from index date  2. Smoking record within +30 to +365 days from index date  3. Smoking record earlier than -365 days from index date  4. Smoking record greater than +365 days from index date |
| --- | --- |
| Alcohol algorithm | We searched each person’s CPRD clinical and additional files for Read codes indicating alcohol intake, CPRD therapy files for antabuse therapy prescriptions, and HES secondary care data for ICD-10 codes indicating alcohol intake. We combined these data and created three levels of alcohol intake: (1) non-drinker, (2) ex-drinker, (3) current drinker. We recoded people whose most recent alcohol intake code indicated non-drinker, but had a history of drinking as ex-drinkers.  We prioritised data indicating alcohol intake in the following order:  1. Alcohol record within -365 to + 30 days from index date  2. Alcohol record within +30 to +365 days from index date  3. Alcohol record earlier than -365 days from index date  4. Alcohol record greater than +365 days from index date |
| Body-mass index (BMI) algorithm | We searched each person’s CPRD clinical and additional files for weight and height measurements recorded by the GP. Key decisions included:   - Allowing heights to carry forward from age 16, updated with a new height entered by the GP. - Dropped height or weight measurements if 3+ were entered on the same day. - Dropped weights <2kg or heights <4 feet or >7 feet. - If 2 height or weight measures on the same day, we took the mean value unless height difference was >5cm or weight difference was >1kg. - Dropped BMI if >200 or <5 kg/m^2^. - Prioritise BMI calculated using weight and height entered by GP. Use BMI entered by GP if height and weight missing, and BMI is within 5-200kg/m^2^ acceptable range.   We prioritised BMI calculated from values entered by the GP in the following order:  1. BMI record within -365 to + 30 days from index date  2. BMI record within +30 to +365 days from index date  3. BMI record earlier than -365 days from index date  4. BMI record greater than +365 days from index date |
