# Supplementary material for: Adverse outcomes after partner bereavement in people with reduced kidney function: Parallel cohort studies in England and Denmark
Source: PLoS One. 2021 Sep 23;16(9):e0257255. doi: 10.1371/journal.pone.0257255 (PMC8460004; doi:10.1371/journal.pone.0257255)
Supplement: S6 Methods — (DOCX) [file pone.0257255.s012.docx]

### **S6 Methods. Comorbidity codelists (ICD-8 or ICD-10) - Denmark**

| **Disease** | **ICD-8** | **ICD-10** |
| --- | --- | --- |
| Myocardial infarction | 410 | I21;I22;I23 |
| Other ischaemic heart disease (excluding myocardial infarction) | 411-414 | I20; I24; I25; Z95.5 |
| Congestive heart failure | 427.09; 427.10; 427.11; 427.19; 428.99; 782.49 | I50; I11.0; I13.0; I13.2 |
| Peripheral vascular disease | 440; 441; 442; 443; 444; 445 | I70; I71; I72; I73; I74; I77 |
| Cerebrovascular disease | 430-438 | I60-I69; G45; G46 |
| Dementia | 290.09-290.19; 293.09 | F00-F03; F05.1; G30 |
| Chronic pulmonary disease | 490-493; 515-518 | J40-J47; J60-J67; J68.4; J70.1;  J70.3; J84.1; J92.0; J96.1; J98.2; J98.3 |
| Connective tissue disease | 712; 716; 734; 446; 135.99 | M05; M06; M08; M09;M30;M31;  M32; M33; M34; M35; M36; D86 |
| Peptic ulcer disease | 530.91; 530.98; 531-534 | K22.1; K25-K28 |
| Liver disease | 571; 573.01; 573.04; 070.00; 070.02; 070.04; 070.06; 070.08; 573.00; 456.00-456.09 | B18; K70.0-K70.3; K70.9; K71; K73; K74; K76.0; B15.0; B16.0; B16.2; B19.0; K70.4; K72; K76.6; I85 |
| Diabetes (type 1 or 2) | 249-250 | E10-E11 |
| Non-hematological malignancy | 140-194; 195-198; 199 | C00-C75; C76-C80 |
| Hematological malignancy | 204-207;  200-203; 275.59 | C91-C95;  C81-C85; C88; C90; C96 |
| Acute Kidney Injury (AKI) |  | N17 |
| Hypertension | 400-404 | I10-I12 |
