# Supplementary material for: Adverse outcomes after partner bereavement in people with reduced kidney function: Parallel cohort studies in England and Denmark
Source: PLoS One. 2021 Sep 23;16(9):e0257255. doi: 10.1371/journal.pone.0257255 (PMC8460004; doi:10.1371/journal.pone.0257255)
Supplement: S7 Methods — (DOCX) [file pone.0257255.s013.docx]

### **S7 Methods. Renal Replacement Therapy (RRT) codelist - Denmark**

| **Condition** | **Operation codes** | **ICD-10** | **Treatment code** |
| --- | --- | --- | --- |
| Any renal replacement therapy (RRT) | 57480; 57490; KKAS | Z940; T861; DZ992; | BJFD0; BJFD2 |
| Kidney transplant | 57480; 57490; KKAS | Z940; T861; |  |
| Dialysis (acute) |  |  | BJFD0 |
| Dialysis (chronic) |  | DZ992; | BJFD2 |
