# Supplementary material for: Adverse outcomes after partner bereavement in people with reduced kidney function: Parallel cohort studies in England and Denmark
Source: PLoS One. 2021 Sep 23;16(9):e0257255. doi: 10.1371/journal.pone.0257255 (PMC8460004; doi:10.1371/journal.pone.0257255)
Supplement: Attachment — Submitted filename: Response to Reviewers.docx [file pone.0257255.s014.docx]

Dear Dr Remuzzi and Editorial Team,

We thank the Editors and Reviewers for your time and effort devoted to reviewing our manuscript. Below you will find our point-by-point response to your comments. We believe that the changes have improved our manuscript, and we hope that you will find them satisfactory.

As stated in our cover letter, we do not wish to make changes to the funding section of our submission. More details are provided in the point-by-point response below (Editor comments, point 2).

Sincerely,

Patrick Bidulka, Søren Viborg Vestergaard (on behalf of all authors)

| **No** | **Reviewer’s comment** | **Author’s response** | **Location in revised manuscript (page)** |
| --- | --- | --- | --- |
| **Editor** |  |  |  |
|  | 1. Please ensure that your manuscript meets PLOS ONE's style requirements, including those for file naming. | Thank you for taking the time to review our manuscript.  We have changed the name of the files, and checked that the manuscript meets the style requirements. Please let us know if there are any further edits we can make to the manuscript. |  |
|  | 2. Thank you for stating the following in the Acknowledgments Section of your manuscript:  "This research was funded in whole or in part by the Wellcome Trust (205039/Z/16/Z)."  We note that you have provided funding information that is not currently declared in your Funding Statement. However, funding information should not appear in the Acknowledgments section or other areas of your manuscript. We will only publish funding information present in the Funding Statement section of the online submission form.  Please remove any funding-related text from the manuscript and let us know how you would like to update your Funding Statement. Currently, your Funding Statement reads as follows:  "The Danish analyses were partly funded by the Beckett Foundation. SML is funded by a Wellcome Senior Clinical Fellowship in Science (205039/Z/16/Z). For the purpose of Open Access, the author has applied a CC BY public copyright license to any Author Accepted Manuscript (AAM) version arising from this submission. The funders had no role in study design, data collection and analysis, decision to publish, or preparation of the manuscript."  Please include your amended statements within your cover letter; we will change the online submission form on your behalf. | Thank you for pointing this out.  We have removed mention of funding from the acknowledgements section of the manuscript.  We do not require any edits to the funding section of the manuscript. We have also indicated this in the cover letter attached with this re-submission. | Acknowledgements, page 21, lines 369-372 |
|  | 3. Please include your full ethics statement in the ‘Methods’ section of your manuscript file. In your statement, please include the full name of the IRB or ethics committee who approved or waived your study, as well as whether or not you obtained informed written or verbal consent. If consent was waived for your study, please include this information in your statement as well. | We have added the ethics statement to the end of the methods section. We have also added the reason for not seeking informed consent from participants in England. We hope the reviewer finds this satisfactory. | Methods, page 10, lines 194-201 |
|  | 4. Please upload a new copy of Figures 1 and 2 as the detail is not clear. | Thank you. We have uploaded the figures as a .tif file instead of .jpg as was submitted previously. We have also uploaded them into Pace to ensure they meet the correct formatting requirements. While they appear low resolution in the PDF, when you click on the link to download the image they appear high resolution. We hope the downloadable figures are now the right resolution.  If they are still not satisfactory, please let us know. | Figures 1 and 2 |
|  | 5. Please include captions for your Supporting Information files at the end of your manuscript, and update any in-text citations to match accordingly. | We have renamed all of the components of the supplementary file to match the journal naming requirements. We have also added the list of supplementary components to the end of the manuscript. | Page 24, lines 493-514 |
|  | 6. Please review your reference list to ensure that it is complete and correct. If you have cited papers that have been retracted, please include the rationale for doing so in the manuscript text, or remove these references and replace them with relevant current references. Any changes to the reference list should be mentioned in the rebuttal letter that accompanies your revised manuscript. If you need to cite a retracted article, indicate the article’s retracted status in the References list and also include a citation and full reference for the retraction notice. | We believe our reference list is complete and correct. Thank you for your comment. | References, line 375 |
| **Reviewer 1** |  |  |  |
|  | This is an interesting manuscript that investigates the association between partner bereavement and hospitalization of CVD and AKI, and death in people with reduced kidney function. The results suggest that partner bereavement was associated with an increased risk of CVD hospitalization, AKI hospitalization, and death in both England and Denmark. The authors provide a clearly written explanation and rationale of the topic and a description of the participants’ selection and outcome assessment. The methods are well detailed and results are professionally presented, providing new evidence on this topic. The study is well designed and presented, while there were some minor issues that need to be addressed: | Thank you for taking the time to review our manuscript. We appreciate the helpful comments you have provided to strengthen the quality of our work. |  |
|  | 1. In Denmark cohort, the information of baseline eGFR did not offer, which limits the effect of CKD stages on the outcomes. The selection of participants was based on the hospital-diagnosed CKD. Could you explain why you do not have the data of eGFR in your cohort? | We agree that eGFR on the Danish cohort would be appreciated. Unfortunately, this was not possible in the current study, as we nested the study in an existing database established for examining the effect of bereavement in chronic diseases. It was not feasible to add eGFR to this database for two reasons. First, Danish laboratory data are stored in another system administered by The Danish Health Data Authority than the one used for this study which is administered by Statistics Denmark. Second, only a subset of this bereaved cohort would be covered by the laboratory database, as this is only nationwide since 2014.  To make this more clear in our manuscript, we have added the sentence “eGFR data were not available in the data sources we used in Denmark” in the covariates section (methods, page 8, line 164). | Methods, page 8, line 164 |
|  | 2. Both of the cohorts included the information of RRT. It might be interesting to see whether RRT affects the outcomes. You can adjust it in your Cox model. | Thank you for this relevant suggestion. To examine if differences in proportion of persons with prior RRT had any effect on the outcomes, we adjusted for any RRT prior to the index date in the Danish cohort. We found that the overall findings changed nothing or little with HR of CVD being unchanged (1.10 [1.04-1.17]), HR of AKI being 1.36 [1.17-1.57] instead of 1.36 [1.17-1.58], and HR of death being 1.21 [1.15-1.26] instead of 1.20 [1.15-1.25]. These minimal differences seen in the model adjusted for RRT may be explained by the fact that the covariate is well balanced in the bereaved and non-bereaved groups in the Danish cohort (12.1% and 12.6%).  The UK cohort included very few persons with prior RRT recorded in primary care (0.4% in both bereaved and non-bereaved groups). We chose not to include prior RRT in our model since the prevalence of prior RRT is so low in the English cohort, meaning we have issues of sparse data for this potential confounder. However, we have re-run our models, since as the reviewer points out, this is a relevant potential confounder. . After including prior RRT as a covariate in the adjusted models, we observed no changes in the hazard ratio point estimates for all three main outcomes and minor changes (by maximum 0.01) in the 95% CI. The newly adjusted estimates are as follows: composite CVD (HR 1.06, 95% CI 1.01-1.11); AKI (HR 1.20, 95% CI 1.11-1.30); and death (HR 1.10, 95% CI 1.06-1.13).  To keep analyses between the two countries as similar as possible, we kept the previous version of the analyses without adjusting for prior RRT. We are of course willing to discuss including RRT in the manuscript if needed. |  |
|  | 3. In Table 1, the p-value is missing, so it is difficult to compare the bereaved and the comparison group. Could you explain why did not show the p-value? | Thank you. We chose not to include p-values in our baseline tables and we prefer not to do so for several reasons. P-values are used in hypothesis testing, and our hypothesis did not concern differences in the baseline characteristics [1, 2]. The STROBE guidelines advises not to include significance tests in descriptive tables [3]. We used clinical subject matter knowledge to select relevant confounders to adjust for in the Cox model, regardless of whether distribution of characteristics was statistically different between cohorts at baseline [3]. We are concerned that placing emphasis on the arbitrary threshold value of p<0.05 to interpret baseline characteristics is inappropriate when the main result of our study is the multivariable models. We believe this is in keeping with other articles published in PLOS ONE but are happy to be advised if this is incorrect. |  |
|  | 4. In the abstract, when you present the information of the rate of CVD, AKI, and death, please add the unit (1000 person-year?). | Thank you for noticing. We have added the unit as suggested one page 2, line 37. | Page 2, line 37 |
|  | 5. In the Result section line 222, you introduced a new term named incidence rate (IR) of CVD. Is it the same meaning as the rates of hospitalizations with CVD? It might be confusing, please keep using the same term throughout the manuscript. | Thank you for this pertinent comment. We have now replaced the term incidence rate (IR) with “rates” throughout the manuscript. Changes can be seen in the results section, lines 223, 227, 242, 244. | Results, lines 223, 227, 242, 244 |
|  | 6. The Figures are not clearly seen, please add high-resolution figures. | Thank you. We have uploaded the figures as a .tif file instead of .jpg as was submitted previously. We have also uploaded them into Pace, a recommended website by the journal, to ensure they meet the correct formatting requirements. While they appear low resolution in the PDF, when you click on the link to download the image they appear high resolution. We hope the downloadable figures are now the right resolution.  If they are still not satisfactory, please let us know. | Figures 1 and 2 |

**REFERENCES**

1.	Palesch YY. Some common misperceptions about P values. Stroke. 2014;45(12):e244-6. Epub 2014/11/08. doi: 10.1161/strokeaha.114.006138. PubMed PMID: 25378423; PubMed Central PMCID: PMCPMC4245357.

2.	Turkiewicz A, Luta G, Hughes HV, Ranstam J. Statistical mistakes and how to avoid them - lessons learned from the reproducibility crisis. Osteoarthritis Cartilage. 2018;26(11):1409-11. Epub 2018/08/11. doi: 10.1016/j.joca.2018.07.017. PubMed PMID: 30096356.

3.	Vandenbroucke JP, von Elm E, Altman DG, Gøtzsche PC, Mulrow CD, Pocock SJ, et al. Strengthening the Reporting of Observational Studies in Epidemiology (STROBE): explanation and elaboration. Annals of internal medicine. 2007;147(8):W163-94. Epub 2007/10/17. doi: 10.7326/0003-4819-147-8-200710160-00010-w1. PubMed PMID: 17938389.
